# The Impact of the COVID-19 Pandemic on Childhood Obesity and Lifestyle—A Report from Italy

**DOI:** 10.3390/pediatric14040049

**Published:** 2022-09-29

**Authors:** Stefano Palermi, Marco Vecchiato, Sonia Pennella, Anna Marasca, Alessandro Spinelli, Mariarosaria De Luca, Lorena De Martino, Fredrick Fernando, Felice Sirico, Alessandro Biffi

**Affiliations:** 1 Public Health Department, University of Naples Federico II, 80131 Naples, Italy; 2 Med-Ex Medicine & Exercise, Via Vittorio Veneto 108, 00187 Rome, Italy; 3 Sport and Exercise Medicine Division, Department of Medicine, University Hospital of Padova, 35128 Padova, Italy; 4 Department of Translational Medical Sciences, University Federico II, 80131 Naples, Italy

**Keywords:** COVID-19 lockdown, childhood obesity, eating habits

## Abstract

During the COVID-19 lockdown, especially in the first wave of pandemic (March 2020), sedentary lifestyle and calorie intake increase in children became considerably more prevalent. The aim of the present paper was to evaluate changes in children’s weights and nutritional habits during the COVID-19 pandemic in Italy. In this cross-sectional observational study, for 3 years, as part of the corporate wellness program (2019–2021) in Emilia Romagna region of Italy, anthropometric data of Ferrari car company employers’ children were collected, analyzed, and compared. Moreover, at the visit of November 2020, performed after the first wave of the pandemic with the most rigorous lockdown rules in Italy, a questionnaire on nutritional and lifestyle habits was administered. We evaluated 307 children (163 M, 10.1 ± 2.3 mean aged in 2019). A significant increase in BMI percentile in 2020 (65.2) compared to 2019 (49.2) was observed; it was confirmed, albeit slightly decreased, in 2021 (64.5). About one-third of participants reported an increase in consumption of fatty condiments and more than half report an increase in consumption of junk food. Levels of physical activity were still high during the COVID-19 lockdown, while sleeping time was significantly reduced. Our findings alert us to the importance of carefully monitoring eating behaviors in young to avoid the adoption of unhealthy food habits and prevent childhood obesity, especially during the period of COVID-19 lockdown.

## 1. Introduction

Despite the progress made in recent years, Italy is still among the European countries with the highest values of excess weight among school-aged children [1]. Recent data shows that, using International Obesity Task Force criteria, one out of five children is overweight, while the percentage of obese and severely obese are respectively 10% and 2%. These findings are the results of the World Health Organization (WHO) European Childhood Obesity Surveillance Initiative (COSI) conducted in 2019 by *OKkio alla SALUTE* [2], the surveillance system coordinated by *Centro Nazionale per la Prevenzione delle Malattie e la Promozione della Salute*, which has recently been designated a WHO collaborating center on childhood obesity. These data are in line with WHO latest reports about childhood weight evaluation worldwide: over 340 million children and adolescents aged 5–19 were overweight or obese and 39 million children under the age of 5 were overweight or obese in 2020 [3].

Most of the clinical forms of childhood obesity are due to unhealthy eating habits, low physical fitness, high sedentary behavior, and poor sleep standards [4,5]. The prevention and treatment of pediatric obesity and its complications represent crucial objectives [6] and can reduce the costs that the National Health System (NHS) will have to bear for the care and assistance of patients with obesity-associated diseases in adulthood [7]. Furthermore, during 2019, thorough an observational study carried out in a corporate wellness program, Sirico et al. [8] not only confirmed the high percentage of overweight state in children, but also showed that many parents often underestimate their child’s weight. This finding explains how it is essential to pay closer attention to the nutritional status of these children, but also that it is important to conduct educational programs to their families. This is especially true for the child population of Emilia Romagna region of Italy, which has been already investigated in recent years not only by the work of Sirico et al. [9,10]

The coronavirus disease 2019 (COVID-19) pandemic is having deep health, social and economic consequences. Among that, sedentary lifestyle and calorie intake increase have become considerably more prevalent because children often found “refuge” in food: not only to fight boredom, but also to vent frustration of many stationary days at home [4,11,12]. This is especially true for the first wave of the pandemic (March 2020) in which the lockdown resulted in the closure of schools in attendance and group sports activities, and where individual physical activity restrictions were particularly harsh [13,14]. Even if the problem was immediately raised by the Italian NHS, which, in agreement with CREA (Center for Food and Nutrition Research), promptly issued recommendations [4], the increase in childhood obesity during COVID-19 lockdown was unavoidable in Italy [11,12,15,16,17].

Therefore, the aim of the present study was to:

(1) Evaluate changes in anthropometric parameters.
(2) Evaluate changes in nutritional and lifestyle habits.

In a population of children during COVID-19 pandemic in Italy.

## 2. Materials and Methods

The setting of the present study was the “Ferrari Formula Benessere”, a multi-factory yearly corporate wellness program managed by the Med-Ex society, focused on the health primary prevention: it is carried out in the Ferrary company based in Maranello (Mo), in the Emilia Romagna region of Italy. During the program, a period is dedicated to the medical evaluation of employers’ children, who underwent several medical evaluations [8].

Therefore, a convenient sample of consecutive children attending this program was selected as sample size. The eligibility criteria for the inclusion of participants included (a) aged 8–15 years; (b) no history of diseases that could have influences on nutrition habits and weight; (c) having to participate in the “Ferrari Formula Benessere” project in 2019, 2020 and 2021

Our study was divided into two sections:

(1) All subjects performed their annual “Ferrari Formula Benessere” screening visit (November 2019, November 2020 and November 2021). Anthropometric data were collected, analyzed, and compared.
(2) In addition to routine scheduled checks, at the visit of November 2020, performed after the first wave of the pandemic with the most rigorous lockdown rules in Italy, a questionnaire on nutritional and lifestyle habits was administered.

### 2.1. Weight Measurement

During the general and nutritional assessment part of the annual visit, child’s weight and height were recorded by TANITA weight scale (model MC-780MA) and GIMA altimeter (model “Astra”), with the methodology already used in a previous similar study [8,18]. The mean value of three consecutive weight and height measurements was recorded for data analysis. The children’s BMI percentiles are calculated through the AnthroPlus software released by the World Health Organization. According to the classification of children’s weight status [19], each subject was classified as underweight (under the 5th percentile), normal weight (between 5th and 84.9th percentile), overweight (between 85th and 94.9th percentile), or obese (above 95th percentile).

Then, a physician (MV) concluded the anthropometric evaluation and the calculation of BMI percentiles.

### 2.2. Nutritional and Lifestyle Habits Questionnaire

A questionnaire based on the one made by OERSA (*Osservatorio sulle Eccedenze, sui Recuperi e sugli Sprechi Alimentari*) at the Research Centre for Food and Nutrition-CREA in Rome [20] was administered by a physician (SP) in the employees’ children dedicated part of “Ferrari Formula Benessere” program at the 2020 visit, after the first wave of COVID-19 pandemic. The questionnaire included questions aimed at investigating differences in lifestyle and behaviors before and after the first COVID-19 lockdown, divided into 4 sections:

- Eating habits: questions regarding eating habits referred to the consumption of foods belonging to the Mediterranean diet but not only, were present in this section. In addition, questions regarding food education were asked, such as consumption patterns, food choices and nutrition labels comprehension.
- Weight change: each parent was asked for their perceptions of their children’s weight change during the pandemic.
- Exercise: how often and for how long the children practiced exercise before and during COVID-19 lockdown.
- Sleeping time: the amount of time spent sleeping before and during COVID-19 lockdown.

### 2.3. Ethics

All data were collected anonymously, and no incentives were given for completing the study. Moreover, the data collection form specifies that data should be used for scientific purposes, in aggregate form and maintaining the privacy of each specific subject. Data have been treated according to privacy rules and protection. Written informed consent was obtained from the children’s legal guardians. All procedures performed in studies followed the Helsinki declaration and its later amendments or comparable ethical standards.

### 2.4. Statistical Analysis

Analyses were carried out using SPSS software (IBM SPSS Statistics for Windows, Version 26.0. Armonk, NY, USA: IBM Corp). Continuous variables were described using means and SDs, and categorical variables were described using absolute values (n) and percentages (%). The chi-squared test was used to compare percentages [21]. The Shapiro-Wilk test was used as distribution test. BMI was not normally distributed, and the Wilcoxon test was used to compare BMI during the three monitored years. A *p*-value of < 0.05 was considered statistically significant.

## 3. Results

The study group included 307 children, 163 boys and 144 girls. Mean age in 2019 was 10.1 ± 2.3 years.

### 3.1. Weight

**Figure 1:**
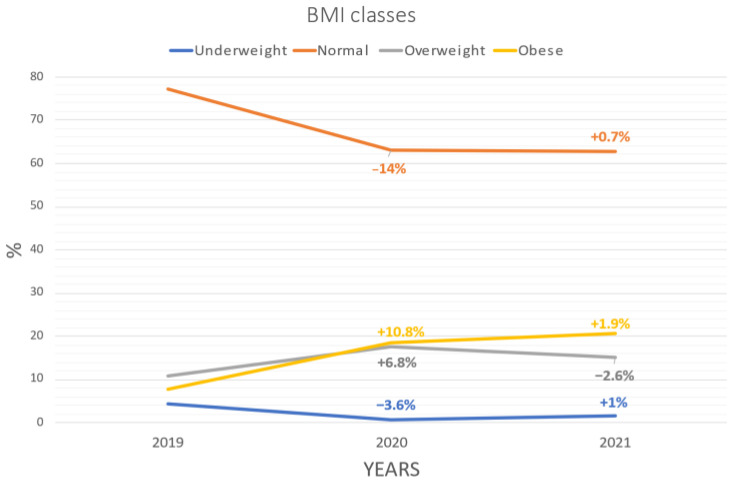
Changes in BMI classes percentages between 2019, 2020 and 2021.

Evaluations in the year 2020 showed a significant increase in BMI percentile (65.2) compared to 2019 (49.2), which was confirmed, albeit slightly decreased, in 2021 (64.5). The number of underweight children decreased (1.6% in 2021) in favor of a significant increase in overweight (15% in 2021) but mostly obese children (20.5% in 2021) (Table 1). Changes in BMI classes percentages are shown in Figure 1

### 3.2. Nutritional and Lifestyle Habits

The responses to the questionnaire are shown in Table 2. Most family groups answered that they maintained similar consumption of all foods during the pandemic, except for the sweet consumption, which was instead reported as increasing in most responses. High percentages reported eating more junk food or snacks and skipping meals and nibbling more often. A low percentage reported spending time reading nutrition labels but a high percentage reported the importance of enjoyed family meals. Most respondents (54.3%) believed that their child’s weight increased during quarantine. The percentage of subjects undertaking physical activity during COVID-19 lockdown was still high (70%), in contrast to sleeping time, which decreased significantly according to our questionnaire: indeed, about three out of four responders reported sleeping less during this period.

## 4. Discussion

Our study monitored a cohort of children of the Emilia Romagna region of Italy for three consecutive years during which the COVID-19 pandemic occurred, by assessing the change in anthropometric parameters and investigating dietary habits before and during the pandemic. The results support the hypothesis that the COVID-19 pandemic can contribute to an increase in children weight through an exacerbation of lots of metabolic risk factors, with dramatic consequences on weight [22].

In fact, during the three-year period, we observed an increase in the number of obese and overweight children and a contemporary reduction of the number of normal-weight and underweight children. This is evident from a significant increase in BMI, both as an absolute value and as a percentile. However, the recorded data confirmed parents’ perception of their children’s weight trends, suggesting awareness of the poor eating habits: this is in disagreement with previous results by Sirico et al. [8], who highlighted a wrong parental perception of children’s weight status.

Such significant increases in a relatively small cohort reflect a substantial change in the eating habits and lifestyle of these children. Indeed, the questionnaire showed an interesting sense of increased consumption of many high-calorie food items. In fact, nearly one-third of participants report an increase in consumption of fatty condiments and more than half report an increase in consumption of sweets. More than half of the participants reported eating junk food and snacks more often and skipping meals during the pandemic. Moreover, even if most of participants described performing physical activity during COVID-19 lockdown, the amount of physical activity performed by the children fell by about 20%: this is in line with similar studies [23,24], that highlighted the reduction of physical activity as one of the worst results of the home-lockdown. Favoring the consumption of junk food, snacking, consuming sweet drinks, and spending most of the time sitting without doing any kind of movement (climbing stairs, walking, playing games) are all determinants of weight gain [25].

Despite the negative impact on anthropometric parameters and unhealthy food consumption, the questionnaire also showed that about one third of the participants increased their consumption of fruit and vegetables, and about one fifth their intake of fish, legumes, and white meat. On one hand, this data suggests that the negative outcomes observed could be primarily associated with a total increase in the daily caloric intake, driven mainly by snacks and desserts, rather than a worsening in the quality of main meals.

Our findings are in line with many other similar studies (Table S3–Supplements Materials), not only conducted in our countries. In Italy, Pietrobelli et al. [12] and Censi et al. [26] found a reduction in time spent in sport activity and an increase in screen time between children during COVID-19 lockdown, and these findings were confirmed all over the world: Androutsos et al. [27] in Greece and Ruiz-Roso et al. [28] in Brazil also stated a reduction in fast food consumption given by the isolation at home, and Philippe et al. [29] in France wrote about an increase in desert intake. Also, an increase in BMI during COVID-19 lockdown was reported by these researchers: Kim et al. [30] found an increase in body weight z-score from 2.0 to 2.2, Yang et al. [31] a prevalence of overweight raising from 21% to 25%, and Woo et al. [32] an uprising of obesity percentages from 23% to 33%. Moreover, in a recent report [33], Kim et al. reported a significant increase in trend of overall obesity in about one million Korean adolescents, even with a slight decrease of the slope during the pandemic.

However, in contrast to these studies, we evaluated a 3-year trend of BMI increase in children that, as shown by our results, was constantly growing independently from 2020 COVID-19 lockdown. Therefore, it is clear that, although this pandemic time negatively influenced childhood weight and nutritional habits, a trend of increasing BMI in children was already evident [34].

Interestingly, positive data also emerged regarding the rediscovery of the importance of family sharing of meals and the importance given to the time it takes to eat meals. The lockdown period generated very different reactions in people. Many people used this forced home restriction period to enjoy the pleasure of a healthy diet and cook more often [35]. This has led to a rediscovery of the importance of conviviality and of family food traditions. Many others, instead, especially in the younger age groups, experienced this period as a malaise, looking for consolation in food. It is well known that the period of stress generated by the lockdown period has caused many worries in people, who have experienced fear and anxiety even in domestic life, dictating an increased consumption of junk foods, rich in fat and sugar, with high calorie intake and without any essential nutrients. Boredom, the lack of a structured day, and the restriction of physical activity have led to an increase in sedentary lifestyle and therefore to a rise in the risk of overweight and childhood obesity, but also of eating behavior disorders. Moreover, in the young, the cancellation of in-person school and sports activities has led to a significant reduction in daily calorie consumption, resulting in an increase in weight and body fat. The excess weight gained during the lockdown period may not be reversible and might contribute to excess adiposity during adulthood [36].

This would suggest increasing programs specifically dedicated to the culture of the Mediterranean diet in children, involving schools, families, and food specialists [37].

Our study suffers from some limitations. Ours is a convenience sample of children, chosen among employers’ sons and daughgters in a defined region of Italy: this does not allow us to generalize our results to the entire population, and this is an important aspect to consider. Regarding nutritional evaluation, we did not perform body impedance or skinfolds analyses, so we are not aware of fat mass and fat free mass of the children: however, we assume that CDC classification of children weight status is accurate enough for children of this age. Moreover, we did not perform the eating habits questionnaire in other years, so that we are not sure that the 2020 answers reflect the other COVID-19 pandemic waves: however, this could be a target for a future study. Regarding the questionnaire, we did not evaluate the possible correlation between anthropometric indicators and answers before and during the pandemic: this could be a possible target for future studies. Finally, we think that it could be very interesting to also assess parents’ BMI to evaluate the impact of COVID-19 on the entire familiar nucleus; moreover, we did not consider the possible association with socio-demographic characteristics of family that could have somehow influenced these results

## 5. Conclusions

The COVID-19 lockdown led to negative changes in childhood nutritional habits and lifestyle, and weight gain was a consequence of that. Results of the present study pointed out these bad consequences in a cohort of children of the Emilia Romagna region of Italy during a 3 years-study period. The increased rates of overweight and obesity in children are directly linked to more health problems, also in their adulthood: this highlights a worrying scenario. Our results support the need for both lifestyle and nutrition education strategies in children to counter the onset of unhealthy habits and a consequent weight increase, especially during the pandemic.

## Figures and Tables

**Table 1:** Comparison of BMI of the same cohort of children (*n* = 307) assessed in 2019, 2020, and 2021. Underweight was considered presenting a BMI below the 5th percentile, overweight between 85th and 94.9th percentile and obese above the 95th percentile.

	2019	2020	2021	*p*
Anthropometric Data				
**Weight (kg)**	30.96 [29.70–32.33]	35.45 [34.05–36.86]	39.63 [38.15–41.10]	<0.001 ^a,b,c^
**Height (cm)**	130.52 [128.89–132.15]	136.29 [134.29–138.11]	142.29 [140.69–143.89]	<0.001 ^a,b,c^
**BMI (kg/m^2^)**	17.58 [17.24–17.91]	18.48 [18.10–18.85]	19.06 [18.67–19.45]	<0.001 ^a,b,c^
**BMI percentile**	49.2 [45.7–52.7]	65.2 [61.9–68.4]	64.5 [61.1–67.8]	<0.001 ^a,b^
**Fat mass (%)**	21.38 [20.63–22.13]	21.83 [21.08–22.57]	23.11 [22.46–23.77]	<0.001 ^a,b^
**BMI classes**				
**Underweight**	13 (4.2%)	2 (0.6%)	5 (1.6%)	
**Normal weight**	237 (77.2%)	194 (63.2%)	193 (62.9%)	
**Overweight**	33 (10.8%)	54 (17.6%)	46 (15.0%)	
**Obese**	24 (7.8%)	57 (18.6%)	63 (20.5%)	

^a:^*p* < 0.05 between 2019 and 2020; ^b:^ *p* < 0.05 between 2019 and 2021; ^c:^ *p* < 0.05 between 2020 and 2021.

**Table 2:** Questionnaire investigating differences in children lifestyle and behaviors before and after the first wave of COVID-19 pandemic.

Eating Habits (Consumption of)			
	MORE	LESS	SAME
olive oil, butter, margarine	60 (30.5%)	11 (5.5%)	126 (64.0%)
fruits	60 (30.5%)	26 (13.2%)	111(56.3%)
vegetables	65 (33%)	28 (14.2%)	104 (52.8%)
white bread	64 (32.5%)	30 (15.2%)	103 (52.3%)
red meat	45 (22.8%)	25 (12.6%)	127 (64.5%)
white meat	38 (19.3%)	17 (8.6%)	142 (72.1%)
sweet beverage	38 (19.3%)	39 (19.8%)	120 (60.9%)
legumes	34 (17.3%)	23 (11.6%)	140 (71.1%)
fish and seafood	44 (22.3%)	33 (16.7%)	120 (60.9%)
desserts	111 (56.3%)	13 (6.6%)	73 (37.1%)
pasta and rice	34 (17.3%)	25 (12.6%)	138 (70.1%)
adding sugar in the milk	3 (1.5%)	11 (5.5%)	183 (92.9%)
water consumption	86 (43.7%)	17 (8.6%)	104 (52.8%)
nibbling	123 (62.4%)	4 (2.0%)	70 (35.5%)
	**NO**	**YES**	
learned to eat meal slowly	62 (31.5%)	135 (68.5%)	
ate more junk food	95 (48.2%)	102 (51.8%)	
ate snack more often	84 (42.6%)	113 (57.4%)	
learned how to read nutrition labels	169 (85.8%)	28 (14.2%)	
skipped meals	74 (37.6%)	123 (62.4%)	
enjoyed family meals	38 (19.3%)	159 (80.7%)	
**Weight increase** **(what do you think?)**			
	**INCREASED**	**NOT** **INCREASED**	
	120 (60.9%)	77 (39.1%)	
**Physical activity** **(did you do?)**			
	**YES**	**NO**	
before COVID-19 lockdown	180 (91.4%)	17 (8.6%)	
during COVID-19 lockdown	139 (70.6%)	58 (29.4%)	
**Sleeping time** **(during COVID-19 lockdown)**			
	**MORE**	**NO MORE**	
	58 (29.4%)	139 (70.6%)	

## Supplementary Materials

The following supporting information can be downloaded at: https://www.mdpi.com/article/10.3390/pediatric14040049/s1, Table S1: Comparison of study about children weight and nutritional habits.
