# Supplementary material for: The Impact of the COVID-19 Pandemic on Childhood Obesity and Lifestyle—A Report from Italy
Source: Pediatr Rep. 2022 Sep 29;14(4):410–8. doi: 10.3390/pediatric14040049 (PMC9589932; doi:10.3390/pediatric14040049)
Supplement: Supplementary file 1 [file pediatrrep-14-00049-s001.zip › pediatrrep-1906224-supplementary.pdf]

Table S1. Comparison of study about children weight and nutritional habits (SUPPLEMENTARY MATERIAL)

**Authors,** **country**	**Sample size**	**Age (years)**	**Impact of lockdown on children's weight**	**Impact of lockdown on children's life style and eating habits**
Pietrobelli et al. [12], Italy	41 participants	13.0 ± 3.1	NA	↑ meals eaten per day ↑ fruit intake ↑ potato chip, red meat, and sugary drink intakes ↓ time spent in sports activities ↑ sleep time ↑ screen time
Kim et al. [30], China	90 participants	12.2 ± 3.4	↑ Body weight Z-score (2.0 ± 0.8 vs. 2.2 ± 0.7) ↑ BMI Z-score (1.9 ± 0.5 vs. 2.0 ± 0.4)	NA
Androutsos et al. [27], Greek	397 participants	7.8 ± 4.1	NA	↑ sleep time ↑ screen time ↓ time spent in sports activities ↑ fruits and fresh fruit juices, vegetables, dairy products, pasta, sweets, total snacks, and breakfast intake ↓ fast food consumption
Yang et al. [31], China	10 082 participants	19.8 ± 2.3	↑ BMI (21.8-22.6) ↑ Prevalence of overweight (21.3%-25.1%)	↑ sedentary time ↑ sleeping time ↑ screen time = physical activity levels

Censi et al. [26], Italy	1027 participants	2-5 years (40%), 6-11 years (60%)	NA	↓ time spent in sports activities<br>↓ adherence to Mediterranean diet<br>↑ screen time
Philippe et al. [29], France	498 participants	7.3 ± 2.2	NA	= snack frequency<br>↑ candy/chocolate, fruit juices, sodas, chips/ salty biscuits, ice creams, pastries/cake/sweet cookies, cream dessert, milks, yoghurt/cheese/quark, fresh and dried fruits, and nuts<br>↓ fruits
Ruiz-Roso et al. [28], Brasil	820 participants	15 mean aged	NA	↑ Legumes, vegetables, and fruit intakes<br>↓ fast food intake<br>↑ fried and sweet food average intakes<br>= sugar-sweetened beverage intakes
Woo et al. [32], Korea	97 participants	11.09 mean aged	↑ BMI (27.3-28.4)<br>↑ Prevalence of severe obesity (23.7%-33%)	↑ screen time<br>↓ time spent in sports activities

BMI: body mass index; NA: not available
